# Supplementary material for: Open-Source Framework for the Visualization, Quantification, and Analysis of 4D Flow and High-Dimensional Magnetic Resonance Images
Source: ArXiv. 2026 Oct 30:arXiv:2610.38948v2. Preprint. [Version 2] (PMC13734148)
Supplement: Supplementary file 1 [file NIHPP2610.38948v2-supplement-1.pdf]

Movie Legends

Movie 1. Retrieval and import of 4D flow CMR data into 3D Slicer. 4D flow series are retrieved from the PACS, organized, and imported with the “Import 4D Flow” module for visualization and analysis. 4D = four-dimensional; CMR = cardiovascular magnetic resonance; PACS = picture archiving and communication system.

Movie 2. 4D flow processing and quantification in SlicerHeart, including phase unwrapping, anatomic segmentation, measurement plane placement, velocity glyph and streamline visualization, and automated quantification of hemodynamic metrics. 4D = four-dimensional.

Movie 3. Free-running cardiac- and respiratory-motion-resolved 5D MRA and 4D flow CMR in a 19-year-old male with truncus arteriosus after repair with VSD closure, RV-PA conduit, and pulmonary valve replacement (illustrative case; same patient as Figure 3). Shown in sequence: (1) volume rendering of the truncal valve from 5D MRA (single respiratory phase) in coronal and axial views; (2) 4D flow CMR with tissue rendering; and (3) tissue rendering with streamlines and with Doppler-like rendering, showing regurgitation through the truncal valve coaptation defect. 4D = four-dimensional; 5D = five-dimensional; CMR = cardiovascular magnetic resonance; MRA = magnetic resonance angiography; RV-PA = right ventricle-to-pulmonary artery; VSD = ventricular septal defect.

Movie 4. Free-running cardiac- and respiratory-motion-resolved 5D MRA and 4D flow CMR in a 5-year-old male with unbalanced atrioventricular canal defect (unbalanced to the left) after atrioventricular valve repair (illustrative case). Shown in sequence: (1) volume rendering from 5D MRA (single respiratory phase) in coronal and axial views; (2) 4D flow CMR with tissue rendering; and (3) tissue rendering with streamlines and with Doppler-like rendering, showing systolic atrioventricular valve regurgitation. 4D = four-dimensional; 5D = five-dimensional; CMR = cardiovascular magnetic resonance; MRA = magnetic resonance angiography.

Movie 5. 4D flow CMR in a 3-year-old female with hypoplastic left heart syndrome after bidirectional Glenn with left pulmonary arterioplasty, Sano shunt ligation, and stent angioplasty of a restrictive atrial septum (illustrative case; same patient as Figure 4). Volume rendering from 4D flow CMR with the stented atrial septal communication marked (red arrow), followed by tissue rendering with streamlines and with Doppler-like rendering, showing flow across the atrial septal communication. 4D = four-dimensional; CMR = cardiovascular magnetic resonance.

Supplementary Material

Supplementary Table S1. Agreement between the framework (3D Slicer) and cvi42 4D flow for all hemodynamic metrics, by Bland-Altman analysis across 108 paired ROIs in 13 patients.

Metric	n	Bias (95% CI)	95% LoA*	Upper LoA (95% CI)	Lower LoA (95% CI)	MAPE	ICC (95% CI)
Forward volume (mL)†	108	−0.1 (−0.3 to 0.1)	2.2 to −2.3	1.4 to 3.0	−3.1 to −1.5	4.8%	0.997 (0.990–0.999)
Effective volume per minute (L/min)†	108	−0.015 (−0.038 to 0.004)	0.21 to −0.24	0.12 to 0.28	−0.34 to −0.13	6.4%	0.994 (0.988–0.997)
Mean velocity (cm/s)†	108	−2.0 (−4.8 to −0.6)	9.8 to −13.9	2.6 to 15.1	−24.3 to −4.2	9.2%	0.795 (0.560–0.973)
Maximum flow (mL/s)†	108	1.2 (−0.5 to 2.8)	16.8 to −14.4	12.0 to 21.5	−18.1 to −10.3	9.0%	0.993 (0.981–0.998)
Cross-sectional area (mm^2)†	108	5.1 (1.5 to 8.5)	46.5 to −36.3	33.0 to 58.4	−47.8 to −24.5	11.1%	0.986 (0.972–0.991)
Backward volume (mL)	108	−0.12 (−0.24 to −0.002)	1.17 to −1.41	0.56 to 1.80	−2.19 to −0.67	2.5%	0.950 (0.709–0.989)
Total volume (mL)	108	−0.2 (−0.5 to 0.0)	2.6 to −2.9	1.2 to 3.7	−4.4 to −1.4	6.1%	0.995 (0.989–0.997)
Net positive volume (mL)	108	1.1 (0.6 to 1.6)	5.1 to −2.9	3.8 to 6.1	−3.2 to −2.4	10.1%	0.991 (0.975–0.995)
Net negative volume (mL)	108	−1.3 (−2.0 to −0.7)	3.2 to −5.8	1.9 to 4.3	−8.1 to −3.5	9.8%	0.811 (0.551–0.924)
Regurgitation fraction (%)	108	0.8 (−0.9 to 1.9)	11.4 to −9.7	6.4 to 15.1	−15.9 to −4.1	2.5‡	0.915 (0.712–0.977)
Volume per minute (L/min)	108	−0.005 (−0.028 to 0.014)	0.19 to −0.20	0.13 to 0.25	−0.26 to −0.13	5.0%	0.996 (0.989–0.998)
Peak velocity (cm/s)	108	−10.0 (−15.7 to −5.9)	30.7 to −50.8	20.5 to 38.7	−68.5 to −33.6	12.7%	0.836 (0.769–0.908)
Maximum pressure gradient (mmHg)	108	−0.8 (−1.4 to −0.4)	3.4 to −5.0	1.5 to 5.1	−7.7 to −2.4	22.4%	0.765 (0.654–0.904)

Bias is the mean difference (framework minus cvi42). *Limits of agreement adjusted for multiple ROIs per patient [42]. †Metrics also reported in Table 2. MAPE was calculated relative to the cvi42 value; for backward, total, and net volumes, relative to the cvi42 forward volume. ‡For regurgitation fraction, the mean absolute difference in percentage points is shown instead of MAPE, because the cvi42 value can be zero. When cvi42 reported no backward flow, its regurgitation fraction was taken as 0%, and the framework value was compared with 0%. Confidence intervals were estimated by patient-level cluster bootstrap (20,000 resamples). 4D = four-dimensional; CI = confidence interval; ICC = intraclass correlation coefficient; LoA = limits of agreement; MAPE = mean absolute percentage error; ROI = region of interest.

Supplementary Table S2. Agreement of the framework (labeled "Slicer") and cvi42 4D flow with 2D phase contrast, by Bland-Altman analysis across the 100 paired ROIs with a 2D phase-contrast forward volume in 13 patients.

Metric	Comparison	n	Bias (95% CI)	95% LoA*	Upper LoA (95% CI)	Lower LoA (95% CI)	MAPE	ICC (95% CI)
Forward volume (mL)	Slicer vs. 2D	100	−2.2 (−3.2 to −1.4)	2.7 to −7.2	1.6 to 3.6	−9.2 to −5.1	21.8%	0.978 (0.934–0.991)
	cvi42 4D vs. 2D	100	−2.1 (−3.0 to −1.3)	2.6 to −6.8	1.5 to 3.5	−8.8 to −4.8	21.3%	0.980 (0.940–0.992)
Backward volume (mL)	Slicer vs. 2D	99	0.28 (−0.03 to 0.64)	3.55 to −2.99	0.98 to 5.44	−4.33 to −1.12	4.0%	0.790 (0.406–0.877)
	cvi42 4D vs. 2D	99	0.37 (0.05 to 0.74)	3.12 to −2.37	0.92 to 4.57	−3.14 to −0.86	3.9%	0.853 (0.589–0.915)
Total volume (mL)	Slicer vs. 2D	99	−2.0 (−2.7 to −1.3)	3.3 to −7.3	1.8 to 5.0	−9.1 to −5.3	21.5%	0.975 (0.930–0.985)
	cvi42 4D vs. 2D	99	−1.8 (−2.5 to −1.1)	3.1 to −6.6	1.7 to 4.7	−8.2 to −4.9	20.5%	0.980 (0.936–0.989)
Regurgitation fraction (%)	Slicer vs. 2D	95	0.9 (−0.6 to 2.5)	17.1 to −15.3	11.3 to 22.3	−18.4 to −10.9	3.7†	0.784 (0.443–0.865)
	cvi42 4D vs. 2D	95	0.2 (−1.6 to 2.5)	15.8 to −15.4	10.0 to 21.4	−18.8 to −11.2	3.6†	0.789 (0.448–0.869)
Effective volume per minute (L/min)	Slicer vs. 2D	100	−0.20 (−0.28 to −0.12)	0.33 to −0.72	0.19 to 0.45	−0.87 to −0.56	23.2%	0.956 (0.905–0.972)
	cvi42 4D vs. 2D	100	−0.18 (−0.26 to −0.11)	0.28 to −0.65	0.15 to 0.40	−0.76 to −0.52	22.4%	0.966 (0.921–0.980)
Peak velocity (cm/s)	Slicer vs. 2D	100	−8.1 (−14.5 to −2.0)	43.0 to −59.3	25.6 to 58.2	−80.8 to −35.1	18.8%	0.777 (0.687–0.894)
	cvi42 4D vs. 2D	100	1.7 (−6.4 to 11.3)	54.4 to −51.0	34.6 to 77.7	−66.7 to −33.2	22.9%	0.809 (0.658–0.903)
Heart rate (bpm)	cvi42 4D vs. 2D	100	4.1 (−2.9 to 13.3)	33.5 to −25.3	7.3 to 54.2	−33.0 to −10.0	7.9%	0.871 (0.715–0.974)

Bias is the mean difference (4D method minus 2D phase contrast). n is lower for backward and total volume (one ROI without a 2D backward volume) and for regurgitation fraction (five ROIs without a 2D value). *Limits of agreement adjusted for multiple ROIs per patient [42]. MAPE was calculated relative to the 2D value; for backward and total volume, relative to the 2D forward volume. †For regurgitation fraction, the mean absolute difference in percentage points is shown instead of MAPE, because regurgitation fraction can be zero. Effective volume per minute from 4D flow was compared with 2D cardiac output (net volume × heart rate). Heart rate was compared for cvi42 4D flow only. Confidence intervals were estimated by patient-level cluster bootstrap (20,000 resamples). 2D = two-dimensional; 4D = four-dimensional; bpm = beats per minute; CI = confidence interval; ICC = intraclass correlation coefficient; LoA = limits of agreement; MAPE = mean absolute percentage error; ROI = region of interest.
